# OsTDL1A binds to the LRR domain of rice receptor kinase MSP1, and is required to limit sporocyte numbers

**DOI:** 10.1111/j.1365-313X.2008.03426.x

**Published:** 2008-05

**Authors:** Xinai Zhao, Justina de Palma, Rowena Oane, Rico Gamuyao, Ming Luo, Abdul Chaudhury, Philippe Hervé, Qingzhong Xue, John Bennett

**Affiliations:** 1 College of Agriculture and Biotechnology, Zhejiang University Hangzhou, China; 2 Plant Breeding, Genetics and Biotechnology Division, International Rice Research Institute Manila, Philippines; 3 Division of Plant Industry, Commonwealth Scientific and Industrial Research Organization Canberra, Australia

**Keywords:** meiosis, apospory, leucine-rich-repeat receptor kinase, RNA interference, rice

## Abstract

Hybrids lose heterotic yield advantage when multiplied sexually via meiosis. A potential alternative breeding system for hybrids is apospory, where female gametes develop without meiosis. Common among grasses, apospory begins in the nucellus, where aposporous initials (AIs) appear near the sexual megaspore mother cell (MeMC). The cellular origin of AIs is obscure, but one possibility, suggested by the *mac1* and *msp1* mutants of maize and rice, is that AIs are apomeiotic derivatives of the additional MeMCs that appear when genetic control over sporocyte numbers is relaxed. *MULTIPLE SPOROCYTES1* (*MSP1*) encodes a leucine-rich-repeat receptor kinase, which is orthologous to EXS/EMS1 in Arabidopsis. Like *mac1* and *msp1*, *exs*/*ems1* mutants produce extra sporocytes in the anther instead of a tapetum, causing male sterility. This phenotype is copied in mutants of *TAPETUM DETERMINANT1* (*TPD1*), which encodes a small protein hypothesized to be an extracellular ligand of EXS/EMS1. Here we show that rice contains two *TPD1*-like genes, *OsTDL1A* and *OsTDL1B*. Both are co-expressed with *MSP1* in anthers during meiosis, but only *OsTDL1A* and *MSP1* are co-expressed in the ovule. OsTDL1A binds to the leucine-rich-repeat domain of MSP1 in yeast two-hybrid assays and bimolecular fluorescence complementation in onion cells; OsTDL1B lacks this capacity. When driven by the maize *Ubiquitin1* promoter, RNA interference against *OsTDL1A* phenocopies *msp1* in the ovule but not in the anther. Thus, RNAi produces multiple MeMCs without causing male sterility. We conclude that OsTDL1A binds MSP1 in order to limit sporocyte numbers. *OsTDL1A*-RNAi lines may be suitable starting points for achieving synthetic apospory in rice.

## Introduction

Hybrids out-perform inbreds under favorable and unfavorable conditions, but they lose heterotic yield advantage when multiplied sexually because of segregation and recombination occurring during meiosis (Bi *et al.*, 2003). A possible alternative breeding system for hybrids is apospory, a form of seed production in which the embryo arises from a nucellar cell without meiosis (Bicknell and Koltunow, 2004). Apospory is common among grasses, including relatives of several major cereal crops (Carman, 1997), but it is unknown in rice and its relatives (Brar *et al.*, 1995). We have initiated a project to develop synthetic apospory for hybrid rice (Bi *et al.*, 2003).

Naturally occurring apospory begins with the appearance of aposporous initials (AIs) alongside the megapore mother cell (MeMC) of the ovule (Bicknell and Koltunow, 2004). Like the MeMC, AIs are enlarged nucellar cells that have the capacity to develop into embryo sacs, but because they do so without undergoing meiosis, the resulting embryo sacs are diploid rather than haploid. Diploid egg cells can develop into embryos by parthenogenesis, i.e. without fertilization, producing progeny that are genetically identical to the maternal tissue (Matzk *et al.*, 2005).

The cellular origin of AIs is uncertain, but our hypothesis is that AIs develop from the same group of nucellar cells that form extra MeMCs when a genetic control limiting MeMC numbers is relaxed. The existence of this control was first suggested by studies on the *multiple archaesporeal cells1* (*mac1*) mutant of maize, which produced extra sporocytes in both ovule (Sheridan *et al.*, 1996) and anther (Sheridan *et al.*, 1999). A similar set of phenotypes was seen in the *multiple sporocytes1* (*msp1*) mutant of rice (Nonomura *et al.*, 2003). As *msp1* was a Tos17 insertion mutant, the insertion site could be cloned and thus identified as a member of the leucine-rich-repeat (LRR) receptor kinase gene family.

MSP1 is closely related structurally and functionally to EXS/EMS1 of Arabidopsis. Both *exs* and *ems1* mutants produce extra sporocytes in the anther (Canales *et al.*, 2002; Zhao *et al.*, 2002). Another Arabidopsis mutant, *tapetum determinant1* (*tpd1*), phenocopied the *exs*/*ems1* mutants, but whereas EXS/EMS1 encoded an LRR receptor kinase, *TPD1* encoded a small, putatively extracellular protein (Yang *et al.*, 2003, 2005). Ma (2005) hypothesized that TPD1 is a ligand for the extracellular LRR domain of EXS/EMS1. Here, we establish that rice contains two close homologues of *TPD1* (*OsTDL1A* and *OsTDL1B*). We examine the sites of expression of these genes relative to *MSP1*, examine the affinity of OsTDL1A and OsTDL1B proteins for the LRR domain of MSP1, and study the effect of RNA interference of *OsTDL1A* on anther and ovule. We confirm that MSP1 and its close paralog MSP1-like1 (MSL1) are structurally the most similar rice proteins to EXS/EMS1.

## Results

### OsTDL1A and OsTDL1B are rice homologs of TPD1 of Arabidopsis

**Figure 1:**
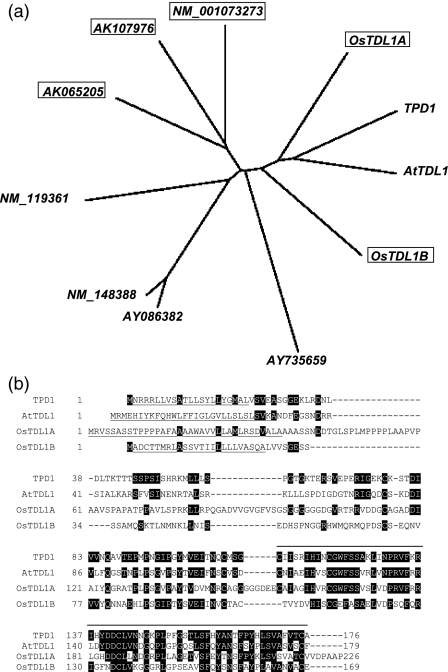
Homologs of Arabidopsis protein TAPETUM DETERMINANT1 (TPD1). a: Unrooted dendrogram based on full-length protein sequences of TPD1, its closest homologs and other related proteins in Arabidopsis and rice. Rice proteins are boxed. Accession numbers for cDNAs: TPD1, AY394849; AtTDL1, BX816721; OsTDL1A, AK108523; OsTDL1B, AK121594. b: Full-length protein alignments for TPD1, AtTDL1, OsTDL1A and OsTDL1B. Sequences were aligned with the BCM Search Launcher program (http://searchlauncher.bcm.tmc.edu/multi-align/multi-align.html). Residues identical to those of TPD1 are highlighted in black. Signal peptides predicted by SignalP-NN are underlined. A line has been placed above the region that is most highly conserved among the four proteins.

The full-length protein sequence of Arabidopsis TPD1 [AAR25553, 176 amino acids (aa)] was used as the query in a tblastn search of the rice genome. We detected two TPD1-like genes and named them OsTDL1A (blast*e*-value: 5 × 10^−27^) and OsTDL1B (blast*e*-value: 1 × 10^−18^). They are located on chromosomes 12 and 10, respectively, and the corresponding full-length cDNAs are AK108523 and AK121594. When their predicted protein sequences (NP_001066753, 226 aa; NP_001064316, 169 aa) were used as tblastn queries of the Arabidopsis genome, the best hits were to TPD1 and another Arabidopsis protein (ABF59206, 179 aa), which we have named AtTDL1. TPD1 is more similar to AtTDL1 (*e*-value: 2 × 10^−35^) than OsTDL1A is to OsTDL1B (*e*-value: 2 × 10^−14^), a conclusion supported by CLUSTALW analysis (Figure 1A). The four proteins cluster separately from the next most similar proteins encoded by the rice and Arabidopsis genomes (the boxed proteins are from rice).

The greater length of OsTDL1 compared with AtTDL1, OsTDL1A and OsTDL1B results principally from four inserts of about 17, 12, 6 and 6 aa in length (Figure 1b). The amino acid identities between TPD1 and any of the other three proteins are highlighted, and are located mainly in the C-terminal half. SignalP-NN software (Nielsen *et al.*, 1997) predicts that all four proteins contain cleavable N-terminal signal peptides (underlined) to target them into the endoplasmic reticulum for export from the cell. Mature molecules of OsTDL1A and OsTDL1B are predicted to contain 191 and 154 aa, respectively.

### Expression of OsTDL1A, OsTDL1B and MSP1 in rice organs

**Figure 2:**
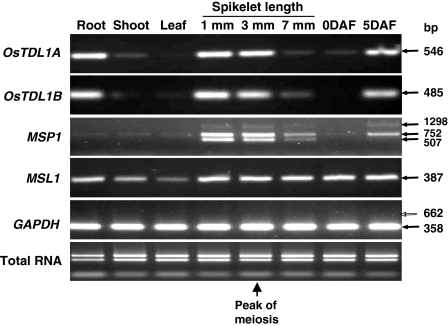
Gene expression in developing ovules and other tissues. RT-PCR for *OsTDL1A*, *OsTDL1B* and *MSP1* genes with RNA from various tissues. Meiosis in the anther and the ovule is most commonly seen in 3-mm spikelets.

*OsTDL1A* and *OsTDL1B* were compared with *MSP1* in terms of spatial and temporal regulation of gene expression (Figure 2). RNA was extracted for RT-PCR from roots, young shoots, flag leaves and spikelets of various stages of development. Before flowering the spikelet stages were defined in terms of spikelet length (1, 3 and 7 mm), with meiosis occurring in anthers and ovules mainly at 3 mm; spikelet samples were also taken at flowering [0 days after flowering (DAF)] and five days later (5 DAF). For each gene, RT-PCR primer sequences were located in exons flanking introns (Figure S1). Single amplicons of the expected size for *OsTDL1A* and *OsTDL1B* (546 and 485 bp, respectively) were generated from RNA of roots and spikelets (1 mm, 3 mm and 5 DAF). Three amplicon sizes were seen for *MSP1*, as was also reported by Nonomura *et al.* (2003). The sizes of these amplicons are consistent with the sizes expected for the fully spliced transcript (507 bp), a transcript in which only the first intron has been removed by splicing (752 bp) and an unspliced transcript (1298 bp), based on the full-length cDNA sequence (AK120933). The smallest amplicon was amplified most strongly from 1- and 3-mm spikelets. The largest amplicon was not a PCR product derived from possible DNA contamination, because the RNA preparations lacked DNA contamination as judged by the failure of the *GAPDH* primers to produce the genomic amplicon (Figure 2, open arrow). We conclude that *MSP1*, *OsTDL1A* and *OsTDL1B* are all expressed in spikelets before and during meiosis.

Unlike *MSP1*, *OsTDL1A* and *OsTDL1B* were also expressed in roots. Yang *et al.* (2003) found that *TPD1* is expressed in the leaves and young seedlings of Arabidopsis, whereas *EXS*/*EMS1* is not expressed in those tissues; roots were not examined. It is not clear why *TPD1* and its rice homologs *OsTDL1A* and *OsTDL1B* are expressed in tissues where transcripts of the receptor kinases are absent. One possibility is that they also interact with proteins other than EXS/EMS1 and MSP1 to regulate processes other than entry into meiosis. A close paralog of MSP1 (MSL1, see Figure S1) was expressed in all tissues examined, including the root (Figure 2), and might interact with OsTDL1A or OsTDL1B. To our knowledge, the expression pattern of *AtTDL1* (Figure 1) has not yet been reported.

### Localization of MSP1, OsTDL1A and OsTDL1B transcripts within spikelets

**Figure 3:**
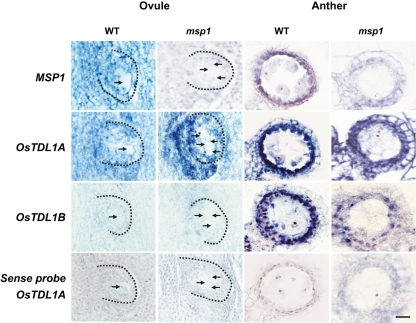
RNA *in situ* hybridization for *MSP1*, *OsTDL1A* and *OsTDL1B* transcripts in the ovule and anther of 3-mm spikelets from wild-type plants (WT) and homozygous *msp1* mutants. Antisense probes were used, except where indicated. Arrows point to megaspore mother cells (MeMCs). Dotted lines indicate the margins of the nucellus. Scale bar: 20 μm.

RNA *in situ* hybridization on 3-mm spikelets indicated that *MSP1*, *OsTDL1A* and *OsTDL1B* were expressed most strongly in the anther, and more weakly in the lemma, palea and basal tissue of the spikelet; transcripts of *MSP1* and *OsTDL1A* were also detected in the ovule, but transcripts of *OsTDL1B* were below the limit of detection. Figure 3 focuses on the expression patterns of the three genes in the ovule and anthers of wild-type cv. Nipponbare and the homozygous *msp1* mutant. All three transcripts were detected with antisense probes (Figure 3), but essentially no transcripts were detected with sense probes (only shown in Figure 3 for *OsTDL1A*). The dotted lines in Figure 3 show the outlines the nucellus, with one or more MeMCs inside (arrowed) and the integuments outside. In wild-type plants, transcripts of *MSP1* and *OsTDL1A*, but not *OsTDL1B*, were clearly seen throughout the ovule (nucellus and integuments), except for the single MeMC. In the homozygous *msp1* mutant, transcripts of both *MSP1* and *OsTDL1B* were difficult to detect in the ovule, but transcripts of *OsTDL1A* were clearly seen throughout the ovule, except for the multiple MeMCs.

The results for *MSP1* agree with those obtained by Nonomura *et al.* (2003), but the results for *OsTDL1A* and *OsTDL1B* are new, and suggest that (i) *OsTDL1A* is much more abundantly expressed in the ovule than *OsTDL1B*, (ii) *OsTDL1A* and *MSP1* tend to be co-expressed throughout the non-sporogenous cells of the ovule, and (iii) neither gene is expressed significantly in the MeMCs. We cannot compare these results with data on *EXS*/*EMS1* or *TPD1* because RNA *in situ* hybridization data for these genes have not yet been reported for the Arabidopsis ovule.

*MSP1*, *OsTDL1A* and *OsTDL1B* were clearly expressed in the anther wall of wild-type plants, but were not expressed in microspore mother cells (MiMCs). In the *msp1* mutant, *OsTDL1A* was strongly expressed in the anther wall, in spite of the absence of the tapetum and other layers (Nonomura *et al.*, 2003). This agrees with data from Arabidopsis, where *TPD1* is expressed in the anther wall in the wild type and in the *ems1* mutant (Yang *et al.*, 2003). The implication is that in rice and Arabidopsis, OsTDL1A and TPD1 are expressed in certain anther wall cells irrespective of whether they differentiate into a tapetum. The difference between our data and those from Arabidopsis (Ma, 2005; Yang *et al.*, 2003) lies in our failure to detect transcription of *OsTDL1A* or *OsTDL1B* in the MiMCs. There is no evidence that *OsTDL1A* resembles *TPD1* in becoming increasingly expressed in MiMCs as the anther matures (Ma, 2005).

We conclude that, in both ovule and anther, *OsTDL1A* and *MSP1* are expressed in similar cell types. In the ovule both genes are widely expressed, but not in the MeMC. In the anther, both genes are expressed in the anther wall. *OsTDL1B* is expressed in the anther wall but not in the ovule.

### Evidence for physical interaction between OsTDL1A and the LRR domain of MSP1

MSP1 is predicted by SignalP-NN, InterProScan and PSORT to be an LRR receptor kinase residing in the plasma membrane, with its LRR domain on the outside of the membrane and its serine/threonine kinase domain in the cytosol. The N-terminal signal peptide would lead *MSP1* co-translationally into the endoplasmic reticulum, but the protein would be retained within the plasma membrane by virtue of a membrane-spanning domain located between amino acids I919 and A934. To examine whether OsTDL1A or OsTDL1B have the potential to interact physically with the LRR domain of MSP1, we employed two-hybrid analysis in yeast cells and bimolecular complementation in onion cells.

#### Yeast two-hybrid analysis

**Figure 4:**
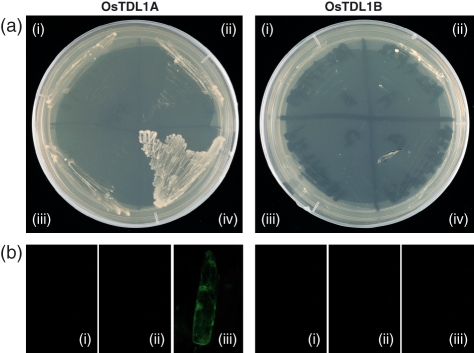
Assay for physical interaction between the leucine-rich-repeat (LRR) domain of MSP1 and either OsTDL1A or OsTDL1B. (a) yeast two-hybrid analysis: i, empty DNA-binding domain with activation domain; ii, empty DNA-binding domain with activation domain of OsTDL1A or OsTDL1B; iii, empty activation domain with DNA-binding domain of MSP1 LRR region; iv, DNA-binding domain of MSP1 LRR region with activation domain of OsTDL1A or OsTDL1B. (b) Bimolecular complementation analysis: i, YN-MSP1Δ+YC; ii, YN+YC-OsTDL1A/OsTDL1B; iii, YN-MSP1Δ+YC-OsTDL1A/OsTDL1B.

The yeast strain used for two-hybrid analysis requires leucine, tryptophan and histidine for growth, but will grow in their absence if a modified *HIS3* gene is activated by co-transformation of the strain with two plasmids that will be maintained by their ability to satisfy the leucine and tryptophan requirements, respectively (James *et al.*, 1996). In this strain, the *HIS3* coding region is under the control of the ADH1 promoter, which binds the GAL4 transcription factor, either as an intact protein or as a complex of its DNA-binding domain and its activation domain. We expressed the DNA-binding domain as a fusion protein with MSP1Δ, a truncated form of MSP1. MSP1Δ contains the first 894 aa of MSP1, including a 34-unit LRR domain, but it lacks the transmembrane domain and the tyrosine kinase domain. Such fusion proteins are directed to the nucleus by the nuclear-targeting signal on the DNA-binding domain. The plasmid carrying the fusion gene provides another gene that satisfies the leucine requirement. The activation domain of *HIS3* was also expressed as a fusion protein with the entire protein molecule of either OsTDL1A or OsTDL1B. This fusion protein was directed to the nucleus by a nuclear-targeting signal on the activation domain, and the plasmid carrying it also satisfied the tryptophan requirement. Physical interaction between MSP1Δ and OsTDL1A or OsTDL1B inside the nucleus of yeast cells would result in yeast growth on medium deficient in leucine, tryptophan and histidine. Transformed yeast grew on this medium when both MSP1Δ and OsTDL1A were co-expressed in the cells, but did not grow when either construct was omitted or when OsTDL1A was replaced by OsTDL1B (Figure 4A).

#### Bimolecular fluorescence complementation in onion cells

This method depends on the reconstitution of an active yellow fluorescent protein (YFP) from two separated domains (Bracha-Drori *et al.*, 2004). The N-terminal domain (YN) was fused with MSP1Δ, and the C-terminal domain (YC) was fused with OsTDL1A or OsTDL1B. Co-expression of the MSP1Δ and OsTDL1A fusions in onion epidermal cells reconstituted the expected green fluorescence, but co-expression of MSP1Δ and OsTDL1B failed to do so (Figure 4b).

We conclude that the two methods used for Figure 4 indicate that OsTDL1A has the capacity to interact physically with the LRR domain of MSP1. Neither method suggested that OsTDL1B has this capacity, at least under the assay conditions in yeast and onion cells. This is consistent with a marked difference in protein sequence between OsTDL1A and OsTDL1B in the region of the protein most highly conserved with TPD1 and AtTDL1 (indicated in Figure 1b).

### RNA interference suppresses OsTDL1A transcript levels in spikelets

**Figure 5:**
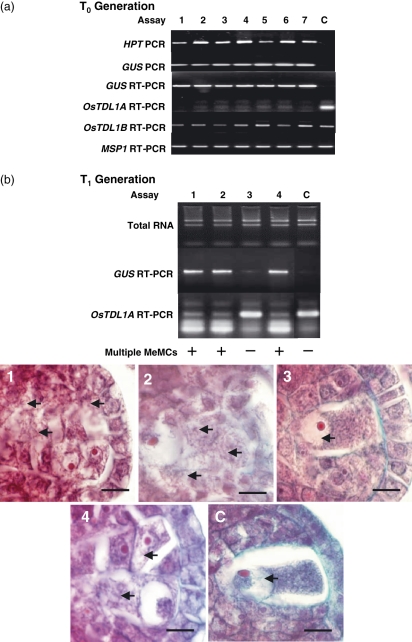
Molecular analysis of *OsTDL1A*-RNAi plants. (a) Spikelets of T_0_ plants: 1–7, independent transformants (4359, 4363, 4374, 4375, 4376, 4378 and 4379, respectively); C, non-transgenic control. DNA and RNA were extracted from 3-mm spikelets and analyzed by PCR for *HPT* and *GUS*, and by RT-PCR for *GUS*, *OsTDL1A*, *OsTDL1B* and *MSP1*. (b) Spikelets of T_1_ progeny from T_0_ transformant 4363: 1–4, T_1_ progeny; C, control plant. RNA was extracted from 3-mm spikelets and analyzed by RT-PCR for *GUS* and *OsTDL1A*. Longitudinal sections of 3-mm spikelets were stained with acridine orange. Leptotene–pachytene–zygotene figures are indicated by arrows. Scale bars: 20 μm.

The proposal that TPD1 might interact physically with EXS/EMS1 (Ma, 2005) was based on genetic evidence that the *tpd1* mutant phenocopies the *exs* and *ems1* mutants in blocking tapetum development and pollen formation in Arabidopsis (Canales *et al.*, 2002; Yang *et al.*, 2003, 2005; Zhao *et al.*, 2002). To check whether downregulation of *OsTDL1A* transcription might phenocopy the *msp1* mutant, we produced RNAi lines in which the *OsTDL1A* gene was targeted. Primary embryogenic calli were co-cultivated with *Agrobacterium* harboring the pANDA vector, in which an *OsTDL1A*-RNAi cassette was under the control of the maize *ubiquitin1* promoter. Transformants were selected and regenerated on hygromycin. Of the 50 independent T_0_ transformants, 47 were positive for the *HPT* gene, and 46 of the *HPT*^+^ plants were also positive for the *GUS* marker located within the RNAi cassette (Miki and Shimamoto, 2004). All *HPT*^+^*GUS*^+^ transformants were positive for expression of the RNAi cassette, as judged by RT-PCR of the *GUS* marker. Figure 5(a) shows PCR amplicons from the *HPT* gene and the *GUS* fragment in seven regenerants, and their absence in a non-transgenic control. All regenerants expressed the RNAi cassette as judged by RT-PCR of the *GUS* fragment. Furthermore, these seven plants showed a high degree of suppression of *OsTDL1A* transcripts. *OsTDL1B* and *MSP1* transcripts were not downregulated in these plants (Figure 5a).

### RNAi of OsTDL1A phenocopies the msp1 mutant in the ovule, but not in the anther

**Figure 6:**
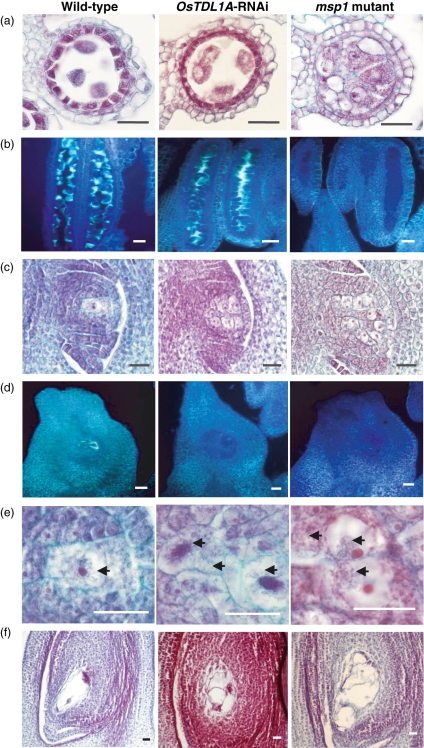
Histology of wild-type cv. Nipponbare, *OsTDL1A*-RNAi plant #4363 and homozygous *msp1* mutant. (a–e) 3-mm spikelets. (f) 7-mm spikelets. (a, c, e, f) Fast green-safranin O staining. (b, d) Aniline blue staining. (a) Transverse section of anther. (b) Longitudinal section of anther. (c–f) Longitudinal sections of ovule. Leptotene–pachytene–zygotene figures are indicated by arrows. Scale bars: 25 μm.

Morphological and histological analyses were conducted on nine independent T_0_*OsTDL1A*-RNAi plants to determine whether they phenocopied the homozygous *msp1* mutant in anther and ovule. The most conspicuous phenotypes of the *msp1* mutant are reduced anther size and complete male sterility (Nonomura *et al.*, 2003), but neither of these phenotypes was seen with *OsTDL1A*-RNAi plants, which showed normal anther size and good fertility for greenhouse conditions. Light microscopy confirmed the lack of *msp1* phenotypes in the anthers of *OsTDL1A*-RNAi plants. Figure 6 presents data obtained with one of the T_0_*OsTDL1A*-RNAi plants (#4363). As in the wild-type, the tapetum was present in #4363 (Figure 6a), and large quantities of callose were associated with the tetrads (Figure 6b). Anthers of *msp1* plants lacked the tapetum, and the locule was packed with MiMCs lacking callose.

By contrast, seven T_0_*OsTDL1A*-RNAi plants phenocopied the *msp1* mutant in the ovule and produced multiple MeMCs. In the case of #4363, the presence of multiple MeMCs (Figure 6c) was accompanied by reduced callose accumulation (Figure 6d), as also seen for the *msp1* mutant. We examined whether the multiple MeMCs were capable of undergoing meiosis by staining for leptotene–pachytene–zygotene figures (Figure 6e). Wild-type ovules contained no more than a single MeMC undergoing meiosis, whereas ovules of *OsTDL1A*-RNAi and *msp1* plants contained several such cells. To estimate the number of multiple MeMCs per ovule, and the fraction of them at pachytene, it was necessary to examine serial sections. *OsTDL1A*-RNAi line #4363 contained about 10 MeMCs per ovule, whereas *msp1* ovules contained about 15 MeMCs. The fraction of MeMCs that were at leptotene–pachytene–zygotene in any given set of serial sections was about 30%. It is not clear whether this percentage reflects a lack of synchrony among putative MeMCs in undergoing meiosis, or a failure of many MeMCs to reach leptotene–pachytene–zygotene stages. Sheridan *et al.* (1996) attributed a low percentage of maize MeMCs undergoing meiosis in such sections to asynchrony.

Although the ovule of the *msp1* mutant contains multiple MeMCs, only one distinct embryo sac is usually observed (Nonomura *et al.*, 2003). However, this embryo sac is surrounded by additional cell-like structures that are absent from wild-type ovules (Figure 6f). These additional structures are also adjacent to the embryo sac of *OsTDL1A*-RNAi ovules (Figure 6f).

### OsTDL1A-RNAi remains effective in the ovule of the T_1_ generation

T_1_ plants derived from T_0_ plant #4363 were examined by PCR for the presence of the construct (*HPT* gene) and the RNAi cassette (*GUS* marker). They were also examined by RT-PCR for expression of the cassette (*GUS* marker) and its effectiveness (downregulation of *OsTDL1A*). Figure 5(b) shows the analysis of three HPT^+^GUS^+^ T_1_ plants and one HPT^−^GUS^−^ T_1_ plant, together with a HPT^−^GUS^−^ non-transgenic control. When RT-PCR was conducted on RNA extracted from 3-mm spikelets of the five plants, only the HPT^+^GUS^+^ T_1_ plants expressed the *GUS* marker and downregulated the *OsTDL1A* gene. Furthermore, only these plants exhibited multiple MeMCs in the ovule. However, all five plants were male-fertile as judged by the normal appearance of the anthers and the production of seeds. We conclude that the *OsTDL1A*-RNAi construct had been transmitted to, and had segregated within, the T_1_ generation, where it was expressed and effective in downregulating *OsTDL1A* transcript levels in spikelets and inducing multiple MeMCs in the ovule without affecting the anther.

### Examination of the failure of OsTDL1A-RNAi to phenocopy msp1 mutants in the anther

We hypothesized that the failure of *OsTDL1A*-RNAi to phenocopy *msp1* in the anther could be caused by (i) a difference between ovule and anther in the control of meiocyte numbers, or (ii) the failure of RNAi to downregulate *OsTDL1A* in the anther. The second explanation is suggested by the report that the maize *ubiquitin1* promoter, used here to drive expression of the RNAi cassette, is not normally expressed in the rice tapetum (Cornejo *et al.*, 1993), which is however a major site of *OsTDL1A* expression. RNA *in situ* hybridization (Figure S2) showed that the tapetum of T_2_ florets in *OsTDL1A*-RNAi line #4363 yielded a hybridization signal with the antisense probe that was substantially higher than the signal obtained with the sense probe. By contrast, in the ovule, RNAi resulted in an antisense hybridization signal similar to that observed with the sense probe, and considerably lower than the antisense signal in the ovule of non-transformed plants. However, examination of three replicate slides for both RNAi plants and control plants, including the slides shown in Figure S2, indicated that the antisense signal for anthers was consistently higher in controls than in RNAi plants. These results suggest that RNAi does reduce the *OsTDL1A* transcript level in the anthers, but not to the extent required to produce a phenotype.

As the maize *ubiquitin1* promoter contains *cis*-elements responsive to heat shock (Streatfield *et al.*, 2004), we explored the possibility that a controlled heat shock might lead *OsTDL1A*-RNAi lines to phenocopy *msp1* in the anther by increasing the degree of downregulation of the RNAi cassette. We measured the effect of a heat shock at the booting stage on spikelet fertility (Figure S3a) and assayed expression of the RNAi cassette by RT-PCR of the *GUS* marker in flag leaves (Figure S3a). We also examined the expression of two representatives of the class-I small heat shock protein genes of rice (Guan *et al.*, 2004). It is evident that 1 h of heat shock at 45°C greatly reduced the fertility of the *OsTDL1A*-RNAi line, whereas it only produced a minor reduction in fertility of the non-transformed control. A heat shock at 40°C (for 0.5 or 3 h) produced a much smaller reduction in fertility in both RNAi plants and non-transgenic controls. Expression of the RNAi cassette (*GUS* marker) in flag leaves was detectable in unstressed transgenic plants, and was upregulated approximately equally by the two intensities of heat shock; expression was absent in stressed and unstressed non-transgenic plants. By contrast, two endogenous heat-shock genes studied previously by Guan *et al.* (2004) were markedly more responsive at 45°C than at 40°C in both RNAi plants and controls, as indicated by the requirement for only 20 cycles of RT-PCR compared with 30 cycles for the *GUS* marker. These results suggest that severe heat shock can cause selective sterility in *OsTDL1A*-RNAi line #4363 by a mechanism requiring interaction between the RNAi cassette and the endogenous heat-shock response.

## Discussion

### OsTDL1A binds to the LRR domain of MSP1

Our data establish that rice contains two close homologs of TPD1, namely, OsTDL1A and OsTDL1B. Only the more similar homolog, OsTDL1A, appears to have a capacity to bind to the LRR domain of MSP1, as judged by two-hybrid analysis in yeast cells and bimolecular complementation in onion cells. OsTDL1B shows no evidence of having a physical affinity for the LRR domain of MSP1. As the LRR domain is extracellular, it is appropriate that the SignalP-NN algorithm predicts that OsTDL1A is secreted from the cell, where it would be able to interact with the LRR domain. It would be intriguing to transform rice with a construct encoding OsTDL1A or OsTDL1B modified with a C-terminal tag (e.g. hexahistidine) to identify their protein partners. This sort of *in vivo* experiment would presumably confirm that OsTDL1A associates with MSP1, and would also throw light on the roles of OsTDL1A and OsTDL1B in tissues such as roots, where MSP1 is not expressed. It may also reveal other proteins associated with MSP1 and OsTDL1A.

The validity of predictions of protein–protein interactions based on yeast two-hybrid analysis was evaluated by Deane *et al.* (2002). They presented four conditions that enhance confidence in the predicted interactions: (i) known functional relationship between the two proteins, (ii) co-expression of the two genes, (iii) existence of evidence for interactions between paralogs of the two proteins, and (iv) agreement between yeast two-hybrid data and data obtained by another method. Our study of OsTDL1A and MSP1 satisfies conditions (i), (ii) and (iv), and may in future satisfy condition (iii). Criterion (i) is satisfied by the fact that *OsTDL1A*-RNAi phenocopies the *msp1* mutant in the ovule. Criterion (ii) is satisfied by the co-expression of the two genes in the ovule and the anther. Criterion (iv) is satisfied by the agreement between yeast two-hybrid data and bimolecular fluorescence complementation data. Future work will examine whether OsTDL1B, a paralog of OsTDL1A, interacts with MSL1, a paralog of MSP1.

### OsTDL1A-RNAi phenocopies the msp1 mutant in the ovule, but not in the anther

*OsTDL1A*-RNAi, when driven by the *ZmUbiquitin1* promoter, downregulates *OsTDL1A* transcript levels without affecting transcript levels for *OsTDL1B* or *MSP1*. These transgenic events copy the phenotype of the *msp1* mutant in the ovule by producing multiple MeMCs and developing an embryo sac that is surrounded by additional structures not seen in the wild type. By contrast, *OsTDL1A*-RNAi lines fail to reproduce the phenotype of *msp1* in the anther, that is, reduced anther size, absence of the tapetum, presence of extra sporocytes in the anther locules, loss of callose around the tetrad, prevention of tetrad maturation and male sterility. The failure to phenocopy *msp1* in the anther may result from the fact that the maize *ubiquitin1* promoter is not normally expressed in the tapetum (Cornejo *et al.*, 1993), which is however a major site of *OsTDL1A* expression. We confirmed that a readily detectable level of *OsTDL1A* transcripts is present in the tapetum of T_2_ plants of RNAi line #4363A, whereas transcripts were not detectable in the nucellus of the ovule in these plants. However, the tapetum *OsTDL1A* transcript level appeared consistently lower in RNAi plants than in controls, suggesting that RNAi was at least partially effective in reducing target transcript levels. A knock-out mutation such as *msp1* is not dependent on a promoter to generate a phenotype, and may therefore give a phenotype in tissues not affected by the RNAi approach. As the tapetum is essential for pollen development (Jung *et al.*, 2005), the *msp1* mutant is male sterile, with pollen development arrested between the leptotene to diakinesis stages (Nonomura *et al.*, 2003), whereas the *OsTDL1A*-RNAi lines remain fertile.

Given that the *ZmUbiquitin1* promoter contains heat-shock elements (Streatfield *et al.*, 2004), we tested whether a controlled heat shock might enhance expression of the RNAi cassette in the rice anther, and so lead *OsTDL1A*-RNAi lines to phenocopy *msp1* in the anther. We did observe much higher heat-induced sterility in *OsTDL1A*-RNAi line #4363 than in the non-transgenic control, but this differential effect was seen only at 45°C, and not at 40°C, and was correlated more with the marked induction of two endogenous heat-shock genes than with the comparatively minor upregulation of expression of the RNAi cassette. Further work will be needed to show whether this interaction between RNAi and the endogenous heat-shock response leads to the loss of the tapetum and pollen callose, as seen in the anthers of the *msp1* mutant.

We note that *exs*/*ems1* (Canales *et al.*, 2002; Zhao *et al.*, 2002) and *tpd1* (Yang *et al.*, 2003, 2005) mutants do not exhibit a phenotype in the ovule in Arabidopsis. Perhaps Arabidopsis and rice differ in their control of MeMC numbers, but it is more likely that the control mechanism is conserved, but that the phenotype is less severe in Arabidopsis than in maize and rice, in terms of the number of extra MeMCs that can form. If that were the case, it may be more difficult to detect the phenotype in the ovule of Arabidopsis.

### Sites of expression and possible roles of TPD1/OsTDL1A and EXS/EMS1/MSP1

We find that *OsTDL1A* and *MSP1* are both expressed strongly in the tapetum of the anther and throughout the ovule (with the exception of the MeMC). In Arabidopsis, *TPD1* and *EXS*/*EMS1* are initially also expressed coincidentally in the inner layers of the developing anthers, but gradually they become more specifically expressed in meiocytes and tapetum, respectively (see the discussion in Ma, 2005). This final expression pattern would be consistent with a model in which TPD1, released from meiocytes, controls the developmental fate of neighboring tapetal cells (or their precursors) by binding to the extracellular LRR domain of EXS/EMS1.

We have not found any similar change in expression sites for *OsTDL1A* and *MSP1* during anther and ovule development. At present, our data suggest that the sites of *OsTDL1A* and *MSP1* expression overlap markedly, with the encoded proteins presumably forming a complex on the outside surface of the cells, but this remains to be established. In the ovule, the zone of overlap between *OsTDL1A* expression and *MSP1* expression is much larger than the zone of cells capable of forming extra MeMCs. This suggests that at least one additional component is required to define the cells that will be induced to become MeMCs upon inactivation of the MSP1-OsTDL1A receptor complex. The weak expression of *MSP1* and *OsTDL1A* in tissues such as the palea and lemma may have no functional consequence if these additional component(s) are not co-expressed in those tissues. Consideration of our data and those of Sheridan *et al.* (1996) for the *mac1* mutant of maize suggests that the additional component(s) may convey positional information defining the L2 layer. This hypothetical component could be extracellular, plasma membrane-bound or cytosolic. The brassinosteroid receptor BRI1 is a close relative of EXS/EMS1/MSP1 (Figure S1), and is known to interact with extracellular brassinosteroid through its LRR domain (He *et al.*, 2000). Within the plasma membrane it activates through homodimerization (Wang *et al.*, 2005), and association with BAK1, a second LRR receptor kinase (Russinova *et al.*, 2004). It is negatively regulated by BK1, a cytosolic protein (Wang and Chory, 2006). Further research on the MSP1/OsTDL1A receptor complex may reveal comparable features. The fact that BRI1 is located in endomembranes as well as the plasma membrane (Geldner *et al.*, 2007) indicates that importance should be given to determining the subcellular location of MSP1 and OsTDL1A, and we should entertain the possibility that OsTDL1A is cleaved to an active form, like CLAVATA3, the ligand of another LRR receptor kinase, CLAVATA1 (Ni and Clark, 2006). It will be particularly interesting to see the identification of the *MAC1* gene, which appears not to be the maize ortholog of MSP1 (Ma *et al.*, 2007). It could be the maize ortholog of OsTDL1A, or it could correspond to yet another component of the ligand-receptor system.

Both *MSP1* (Nonomura *et al.*, 2003) and *OsTDL1A* (X. Zhao, unpublished data) are expressed throughout the nucellus prior to meiosis, whereas the primary MeMC appears not to express these genes, and the extra MeMCs do not appear to express *OsTDL1A*. This result suggests that entry into meiosis and termination of *MSP1* and *OsTDL1A* expression are somehow connected. One possibility is that the termination of *MSP1* and *OsTDL1A* expression is a consequence of the entry into meiosis; another is that termination of expression is part of the signal leading to meiosis. The second possibility is consistent with the induction of meiosis in a limited number of nucellar cells of the L2 layer by the *mac1* and *msp1* mutations, and *OsTDL1A*-RNAi. A crucial question is the role of the L1 layer in this induction, comparable with the role of the L1 layer in the shoot meristem (Kessler *et al.*, 2006; Lenhard and Laux, 2003).

### Roles of OsTDL1B and AtTDL1

Our data suggest that OsTDL1B does not bind to the LRR domain of MSP1. We may learn more about the function of OsTDL1B from the analysis of *OsTDL1B*-RNAi plants that are currently being developed. One possibility is that OsTDL1B binds to MSL1, a rice paralog of MSP1. The *MSL1* gene is located on chromosome 2, but appears to have been incorrectly annotated in the public databases; we offer a new annotation in Figure S1b, supported by partial sequencing of MSL1 cDNA (Figure S1c). Unlike rice, Arabidopsis appears to have no close paralog of EXS/EMS1 (Figure S1d). However, we reported in Figure 1 that Arabidopsis encodes a close paralog of TPD1, designated here as AtTDL1. It will be interesting to determine whether both TPD1 and AtTDL1 bind to the LRR domain of EXS/EMS1.

### Implications of fertile OsTDL1A-RNAi lines for achieving synthetic apospory in rice

We hypothesized that AIs originate from the same nucellar cells that form extra MeMCs when the control mechanism limiting the number of MeMCs in the ovule is relaxed. In rice, that relaxation may be produced by interfering with the function of MSP1 (Nonomura *et al.*, 2003) or OsTDL1A (this report). However, at least one additional behavioral change is required to produce AIs: the extra MeMCs must bypass meiosis and enter directly into diploid mitosis to produce aposporous embryo sacs. We suggest that *OsTDL1A*-RNAi lines are superior to the *msp1* mutant as a platform for producing this behavioral change because of their fertility, which is manifested in both the T_0_ and T_1_ generations. Although our results indicate that the *OsTDL1A*-RNAi line #4363 is more susceptible to severe heat shock (45°C) than the non-transformed control, it is unlikely that the temperatures normally encountered in the field will jeopardize the use of the RNAi approach for achieving apomixis in hybrid rice.

Our approach to synthetic apospory has been influenced by studies on Kentucky Bluegrass (*Poa pratensis*), where sexual and apomictic lines differ at five genetic loci (Matzk *et al.*, 2005). These loci are termed apospory prevention (*APV*, *apv*), apospory initiation (*AIT*, *ait*), megaspore development (*MDV*, *mdv*), parthenogenesis prevention (*PPV*, *ppv*) and parthenogenesis initiation (*PIT*, *pit*), where the lower case allele is recessive. Full sexuality corresponds to one allele set (*APV*, *ait*, *MDV*, *PPV*, *pit*), whereas full apospory corresponds to another (*apv*, *AIT*, *mdv*, *ppv*, *PIT*). Matzk *et al.* (2005) suggested that *APV* may correspond to *PpMAC1*. Our results and those of Nonomura *et al.* (2003) suggest that *APV* may be *PpMSP1* or *PpTDL1A*, with *apv* being a mutant allele relaxing control over MeMC numbers. We are currently examining candidate genes to play the role(s) of *AIT* and *mdv*.

## Experimental procedures

### Plant materials

Seeds of rice (*Oryza sativa* L. cv. Nipponbare, IRTP number 06669) were obtained from the International Network for the Genetic Evaluation of Rice at the International Rice Research Institute (IRRI, http://www.irri.org/). Plants were grown individually in pots of soil under glasshouse conditions with natural lighting and fertilization as described by Ji *et al.* (2007). For RNA extraction, tissue samples were frozen immediately in liquid nitrogen and stored temporarily at −80°C. The samples included roots and shoots from 10 days after germination (DAG), leaves harvested at 30 DAG, and spikelets harvested at 1, 3 or 7 mm in length (before flowering) and at 0 and 5 DAF.

### Bioinformatics

MSP1, EXS/EMS1 and TPD1 protein sequences were obtained from the NCBI database (http://www.ncbi.nlm.nih.gov), and were used in tblastn searches (http://www.ncbi.nlm.nih.gov/blast) to identify genes encoding closely related proteins in the Arabidopsis and japonica rice genomes. Deduced protein sequences were analyzed for subcellular location using tools available at http://us.expasy.org/tools, together with SignalP-NN (Nielsen *et al.*, 1997) and PredSL (Petsalaki *et al.*, 2006). The deduced amino acid sequences were organized into multiple alignments and phylogentic trees using clustalw (http://clustalw.genome.jp) and TreeView (Page, 1996).

### Microscopy

Spikelets at different stages were fixed overnight in FAA solution [10% (v/v) formaldehyde, 50% (v/v) absolute ethanol, 5% (v/v) acetic acid], dehydrated through a graded ethanol series and embedded using paraffin (Paraplast Plus; Sigma-Aldrich, http://www.sigmaaldrich.com). Serial sections of 5-μm thickness were placed on Fisherbrand® Superforst®/Plus microscope slides (Fisher Scientific, http://www.fishersci.com) and incubated at 45°C for 24 h. Sections were de-waxed in xylene, rehydrated through a graded ethanol series, and stained in fast green and safranin or aniline blue. Sections were viewed under a bright-field microscope (Carl Zeiss, http://www.zeiss.com), supported by Image-Pro Plus 5.1 software (Media Cybernetics, http://www.mediacy.com).

### RNA extraction and RT-PCR

Total RNA was extracted from different tissues by Trizol, according to the instructions from the manufacturer (Invitrogen, http://www.invitrogen.com). RNA was quantified as described by Ji *et al.* (2005). RNA samples were then treated with RNase-free DNase (Promega, http://www.promega.com/) to remove any contaminating genomic DNA. Gene-specific amplification was conducted on transcripts of *MSP1*, *MSL1*, *OsTDL1A*, *OsTDL1B*, a segment of the *Escherichia coli*β-glucuronidase gene (*GUS*) and two endogenous rice heat-shock genes, *Oshsp16.9A* and *Oshsp17.9A*, described by Guan *et al.* (2004). The primer pairs used in reverse transcription-polymerase chain reaction (RT-PCR) were as follows (forward then reverse, written from 5′ to 3′): *MSP1* (ATCTCCAGGTTTTTAGGCTTTACG, CTAGCAGGATGAAAAGCCAGAAAC); *MSL*1 (CATAACTAGCACGGTGCCAC, GATGCTGCAGATTCACCATG); *OsTDL1A* (AACCCTACTACTACTCCTCC, TCATCACGTCCACCGTGTAC); *OsTDL1B* (AGCTTGAGCAAGTATTTGGC, GAAGCCGATACGCTGGAACT); *GUS* (CATGAAGATGCGGACTTACG, ATCCACGCCGTATTCGG); *Oshsp16.9A* (GCTCCTGAAGATGTGATCGG, CCTCAACGAGCAAGAACTAA); *Oshsp17.9A* (GCATCGCCGGCGTGCCGCGTGCGC, CTGACACGACGCGACACACGACTG).

The locations of the primers within the rice genomic sequence are given in Figure S1(a). In each case the primers flanked introns to permit a clear distinction to be made between expected RT-PCR and PCR products, and to identify incomplete splicing. RT-PCR for each gene was performed with SuperScript™ One-Step RT-PCR with Platinum®*Taq* (Invitrogen), according to the manufacturer's instructions. The cytosolic glyceraldehyde-3-phosphate dehydrogenase (*GAPDH*) gene was used as a control for successful amplification and absence of genomic DNA. Primers for the *GAPDH* gene were from Kathiresan *et al.* (2002). RT-PCR products were separated by electrophoresis in 1.5% agarose gels, post-stained using ethidium bromide, and viewed using Gel Doc™ XR System (Bio-Rad, http://www.bio-rad.com).

### RNA in situ hybridization

Templates for RNA probe synthesis were prepared by cloning RT-PCR amplicons into the pGEM®-T Easy vector (Promega). The forward and reverse RT-PCR primers are given below (from 5′ to 3′), and their locations in the genes are illustrated in Figure S1(b): *MSP1* (CTCGTAGCTATAGAGTAACCG, CACTTAGAAACAGGCAAGCAG); *OsTDL1A* (TCGAGTACACCAACTCCTTC, CTGTGAGTGATACTGACATG); *OsTDL1B* (TCGGCTTCAACGACTGTCTG, GTACGTAGCTAATAGGCCAG). The clones were sequenced (Macrogen, http://www.macrogen.com) to determine fidelity and orientation. RNA probes (sense and antisense) were transcribed from the above plasmids using T7 and SP6 RNA polymerases (Promega). Transcription used 11-digoxigenin-UTP instead of UTP. RNA *in situ* hybridization was conducted on ∼ 3-mm spikelets, which were fixed in FAA solution, dehydrated and sectioned as above. Sections were pre-hybridized and hybridized as described by Kathiresan *et al.* (2002) and Ji *et al.* (2005). After hybridization, slides were immersed in stop buffer (2X SSPE), cover slips were removed, slides were washed and exposed for 2 h to anti-digoxigenin antibody that was conjugated to alkaline phosphatase (anti-dig-AP) (Roche, http://www.roche-applied-science.com). Sections were washed twice, spread with AP substrate solution, transferred into a dark humid box and incubated overnight. Enzyme–substrate color reaction was terminated with TE buffer. Sections were dehydrated through a graded ethanol series and mounted. Blue hybridization signals on the tissues were viewed under bright field in a microscope (Carl Zeiss) supported by Image-Pro Plus software.

### Protein–protein interaction studies

#### Construction of plasmids

cDNA molecules corresponding to the longest putative open reading frames (ORFs) of *OsTDL1A* and *OsTDL1B* were obtained by RT-PCR of RNA extracted from 3-mm spikelets. The following primer pairs were used (forward, reverse, from 5′ to 3′): *OsTDL1A* (CCCTACTACTACTCCTCCTC, TTTCCTTGCGGCGATTGACG); *OsTDL1B* (AGCTTGAGCAAGTATTTGGC, GAAGCCGATACGCTGGAACT). The amplicons were cloned into pGEM®-T Easy vector and sequenced. To create *OsTDL1A*, *OsTDL1B* and *MSP1* Gateway Entry clones (Invitrogen), PCR was conducted using gene-specific primers with 5′ extensions corresponding to sequences flanking the *attB* sequence. The PCR templates were the cDNA clones of *OsTDL1A* and *OsTDL1B* and a full-length cDNA clone of *MSP1* (accession number AB103395, which was kindly provided by Dr A. Miyao, National Institute of Agrobiological Sciences, Tsukuba, Japan). However, rather than expressing the entire MSP1 ORF, we created a truncated form of the protein (MSP1Δ) that consists of the first 894 aa of MSP1, that is, the N-terminus and the 34 LRR units, but without the transmembrane and protein kinase domains. The *attB*-flanked PCR products were inserted into the pDONR™ 201 vector in the presence of BP clonase (Invitrogen). The inserts of the Entry plasmids were sequenced bidirectionally using primers targeting the pDONR,vector in order to verify the sequence and the achievement of the desired reading frame. The following primers were used: *OsTDL1A attB1*, GGGGACAAGTTTGTACAAAAAAGCAGGCTTGAGGGTCTCCTCGGCGTCCAG; *OsTDL1A attB2*, GGGGACCACTTTGTACAAGAAAGCTGGGTTCTATGGGGCGGCGGGGTCGACG; *OsTDL1B attB1*, GGGGACAAGTTTGTACAAAAAAGCAGGCTTGGCCGACTGCACTACGATGCGTT; *OsTDL1B attB2*, GGGGACCACTTTGTACAAGAAAGCTGGGTTCTACTCACACGCGACATTAGCT; *MSP1 LRR attB1*, GGGGACAAGTTTGTACAAAAAAGCAGGCTTGGTATCCAATAGTTTCTGGCTTTTCA; *MSP1 LRR attB2*, GGGGACCACTTTGTACAAGAAAGCTGGGTATCCTGCAGCACAATCTGCCAAG.

#### Yeast two-hybrid analysis

The method of James *et al.* (1996), as modified by Luo *et al.* (2000), was followed. Modified pACT2 and pAS2 yeast expression vectors that contain *attR* sites were used to generate the hybrid containing the GAL4 AD (amino acids 768–881) and GAL4 DNA-BD (amino acids 1–147), respectively. Target sequences of *OsTDL1A* and *OsTDL1B* were recombined into the pACT2 vector, and the MSP1 LRR region was recombined into the pAS2 vector, by the LR reaction. Then, pACT2TDL1A and pACT2TDL1B were transformed into PJ69-4A yeast host stain using the lithium acetate procedure (James *et al.*, 1996). Successful introduction was verified by growing yeast on synthetic drop-out medium plates lacking l-leucine. Using the same method, pAS2MSP1 and pACT2TDL1A or pACT2TDL1B were co-transformed into the yeast cells and selected on double-selective medium without l-leucine and l-histidine HCl. Strains showing protein–protein interactions were selected based on activation of the HIS3 reporter gene on plates lacking leucine, tryptophan and histidine in two independent experiments.

#### Bimolecular fluorescence complementation

The method of Bracha-Drori *et al.* (2004) was used. The entry clones pDONR™ 201 TDL1A, pDONR™ 201TDL1B and pDONR™ 201 MSP1 (see above) were also separately recombined into pBiFC GW cC1 and pBiFC GW nC1 vectors in the presence of LR Clonase (Invitrogen), respectively. BiFC vectors and the method used for the bombardment of the recombined plasmid-coated gold particles into onion were all kindly provided by S. Curtin (CSIRO, http://www.csiro.au).

### RNA interference of rice OsTDL1A gene

To generate an RNA interference construct for the *OsTDL1A* gene, gene-specific primers were designed in the 3′ untranslated region. The primers produced a 409-bp amplicon through PCR and the use of the Platinum®*Pfx* DNA polymerase with proof-reading function (Invitrogen). The forward primer contained CACC at the 5′ end for TOPO cloning. Primers used for *OsTDL1A* were as follows (forward, reverse, from 5′ to 3′): CACCGTACACGGTGGACGTGATGA, GAGTGATACTGACATGGGGT. The amplicon was cloned into the Gateway pENTR/D-TOPO cloning vector (Invitrogen), as described by Miki and Shimamoto (2004) and Miki *et al.* (2005). The insert was sequenced for verification, and transferred into the pANDA destination vector by LR recombinase reaction. To allow stem-loop formation in the transcript from the RNAi cassette, the amplicon was inserted into the vector in opposite orientations at *attB1* and *attB2* recombination sites, flanking a partial GUS linker and marker sequence. After the recombinase reaction, the construct was transformed into *E. coli* DH5α cells and recovered from kanamycin-resistant colonies. The correctness of the cassette construction was verified by double digestion using *Sac*I and *Kpn*I, which cut unique restriction enzyme sites in the pANDA vector. Transgenic rice (*O. sativa* L. cv. Nipponbare) plants were produced by *Agrobacterium tumefaciens*-mediated transformation of rice primary embryogenic calli (Toki, 1997). Transformants were selected and regenerated on media containing hygromycin B. Regenerated transgenic rice plants were grown in the IRRI's Confinement Level CL4 Transgenic Greenhouse under natural lighting conditions. Transformation was confirmed by PCR of the hygromycin phosphotransferase gene and the *GUS* marker in leaf samples taken at the vegetative stage of growth. Transcriptional activity of the cassette was established by RT-PCR of the partial *GUS* sequence; its effectiveness in downregulating *OsTDL1A* transcript levels and its impact on *OsTDL1B* and *MSP1* transcript levels were assessed by RT-PCR, using RNA extracted from 3-mm spikelets of T_0_ and T_1_ plants.

### Heat shock

Nipponbare (non-transgenic control) and *OsTLD1A*-RNAi plants at the booting stage were exposed to heat shock at 40°C for 0.5 or 3 h, and at 45°C for 10 min or 1 h in a Biotron Growth Cabinet, Model LPH-200-RD (NK Systems; Nippon Medical and Chemical Instruments; http://www.nihonika.co.jp). Humidity was set at 70%. Flag leaves were harvested 0.5 or 3 h after each treatment, and RNA was extracted and RT-PCR was conducted on samples. At grain maturity spikelet fertility was recorded.

## Supplementary material

The following supplementary material is available for this article online:

Appendix S1 Annotation of MSL1.

Figure S1 Exon-intron structure of OsTDL1A, OsTDL1B and MSP1 and partial structure of MSL1.

Figure S2 Use of RNA in situ hybridization to detect OsTDL1A transcripts in the ovule and anther of 3 mm spikelets (stage of maximum meiosis) of non-transgenic plants (control) and T2 plants of OsTDL1A-RNAi line #4363.

Figure S3 Effect of heat shock (HS) at the booting stage on spikelet fertility and gene expression in cv Nipponbare (control) and T2 plants of OsTDL1A-RNAi line #4363.

This material is available as part of the online article from http://www.blackwell-synergy.com
