# Supplementary material for: OsTDL1A binds to the LRR domain of rice receptor kinase MSP1, and is required to limit sporocyte numbers
Source: Plant J. 2008 May;54(3):375–87. doi: 10.1111/j.1365-313X.2008.03426.x (PMC2408674; doi:10.1111/j.1365-313X.2008.03426.x)
Supplement: Appendix S1 — Annotation of MSL1. [file tpj0054-0375-SD1.doc]

**APPENDIX S1**

**1. Annotation of MSL1**

The inability of OsTDL1B to bind to the LRR domain of MSP1 (Figure 4) raised the possibility that OsTDL1B might bind to a paralogue of MSP1 in rice. We used TBLASTN analysis to scan the rice genome and found a paralogous gene encoding a protein which we named MSP1-like1 (MSL1). Whereas *MSP1* is located on chromosome 1, *MSL1* is located on chromosome 2. This gene has been included in rice genome annotation but the predicted protein (XM_464445) does not have a similar N-terminus to MSP1 (and EXS/EMS1 of Arabidopsis); the PSORT program predicts it to be targeted to the thylakoid membrane. We examined the annotation and found that the gene was predicted to consist of a coding region of 1413 amino acids, separated into two exons by a 2 kb intron. The first exon was predicted to encode 101 aa and the second exon was predicted to encode 1312 aa. This contrasts with the *MSP1* gene, which possesses an intron-free coding sequence specifying a protein of 1294 aa and two introns in the 5’-untranslated region (Nonomura et al., 2003, and Figure S1A).

We reviewed the structure of the genome for 1 kb upstream from the 3’-end of the predicted intron of *MSL1* using GenScan (<http://genes.mit.edu/GENSCAN.html>), which made an alternative prediction. GenScan predicted two exons separated by an intron, but the intron would be only 689bp in length, and the first exon would encode only 4 aa, while the second exon would encode 1312 aa (Figure S1B). We tested this prediction by sequencing of an RT-PCR product amplified using primers flanking the predicted intron (Figure S1B). The sequence agreed with the prediction in terms of both the upstream exon and the location of the intron (Figure S1C).

When the subcellular targeting of the revised MSL1 protein was examined, all algorithms predicted that it would be transported to the plasma membrane, like MSP1. Furthermore, when we used InterProScan (http://www.ebi.ac.uk/InterProScan/) to determine the organization of LRR units in MSP1, in XM_464445 and in our new annotation of MSL1, it became clear that the 40-aa LRR closest to the N-terminus was at exactly the same distance from the N-terminus in MSP1 and the re-annotated MSL1 (22 residues) while the N-terminal LRR in XM_464445 was 119 residues distant. We conclude that our new annotation for MSL1 is correct.

**2.** **Examination of the failure of *OsTDL1A*-RNAi to phenocopy *msp1* mutants in the anther.**

The text related to Figures S2 and S3 is located in the last section of the Results.

**Legend for Figure S2**

Use of RNA *in situ* hybridization to detect *OsTDL1A* transcripts in the ovule and anther of 3 mm spikelets (stage of maximum meiosis) of non-transgenic plants (control) and T_2_ plants of *OsTDL1A*-RNAi line #4363. AS: *OsTDL1A* anti-sense probe, S: *OsTDL1A* sense probe. Arrows indicate the tapetum. Bar: 20 μm.

**Figure S3**

Effect of heat shock (HS) at the booting stage on spikelet fertility and gene expression in cv Nipponbare (control) and T_2_ plants of OsTDL1A-RNAi line #4363. A: Plants were exposed at booting to either 0.5h or 3h at 40^0^C or 10 min or 1 h at 45^0^C. Spikelet fertility was measured at grain maturity as filled-grain percentage. B: Flag leaves were harvested either 0.5h or 3h after the end of heat shock (post-HS), or without heating shock (C), and RNA was extracted for RT-PCR assay of transcript levels for the RNAi cassette (using the *GUS* marker) or two endogenous small heat shock protein genes, *Oshsp16.9A* and *Oshsp17.9A*. The annealing temperatures and cycle numbers for the three transcripts were as follows: *GUS*, 56^0^C, 30 cycles; *Oshsp16.9A*, 58^0^C, 20 cycles; *Oshsp17.9A*, 60^0^C, 20 cycles.
