# Supplementary figures and images for: OsTDL1A binds to the LRR domain of rice receptor kinase MSP1, and is required to limit sporocyte numbers
Source: Plant J. 2008 May;54(3):375–87. doi: 10.1111/j.1365-313X.2008.03426.x (PMC2408674; doi:10.1111/j.1365-313X.2008.03426.x)

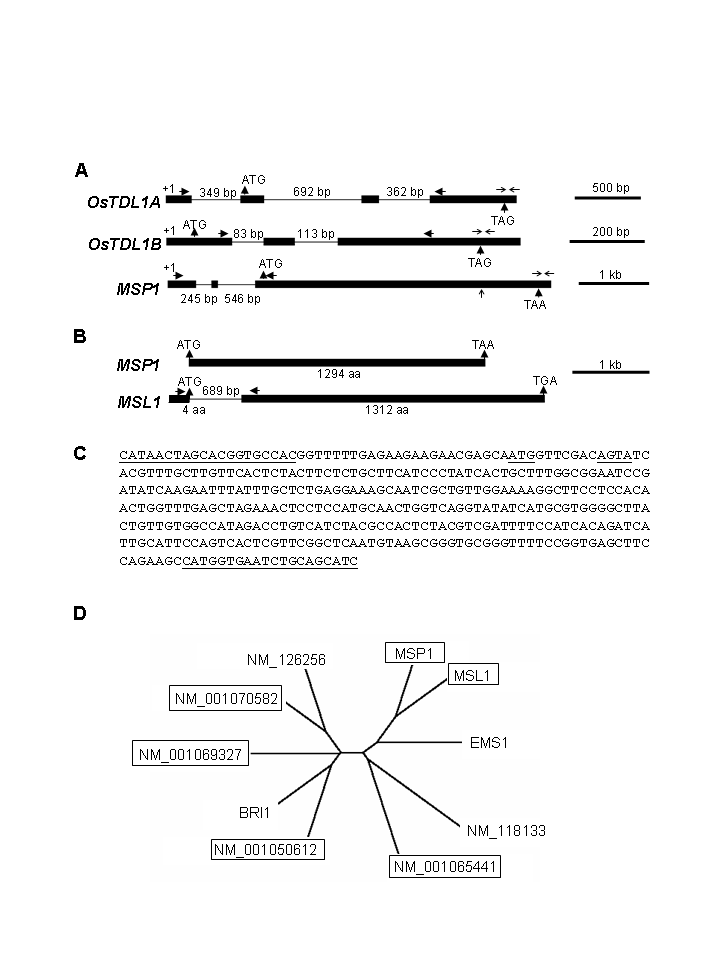

Supplement: Figure S1 — Exon-intron structure of OsTDL1A, OsTDL1B and MSP1 and partial structure of MSL1. [file tpj0054-0375-SD2.tif]

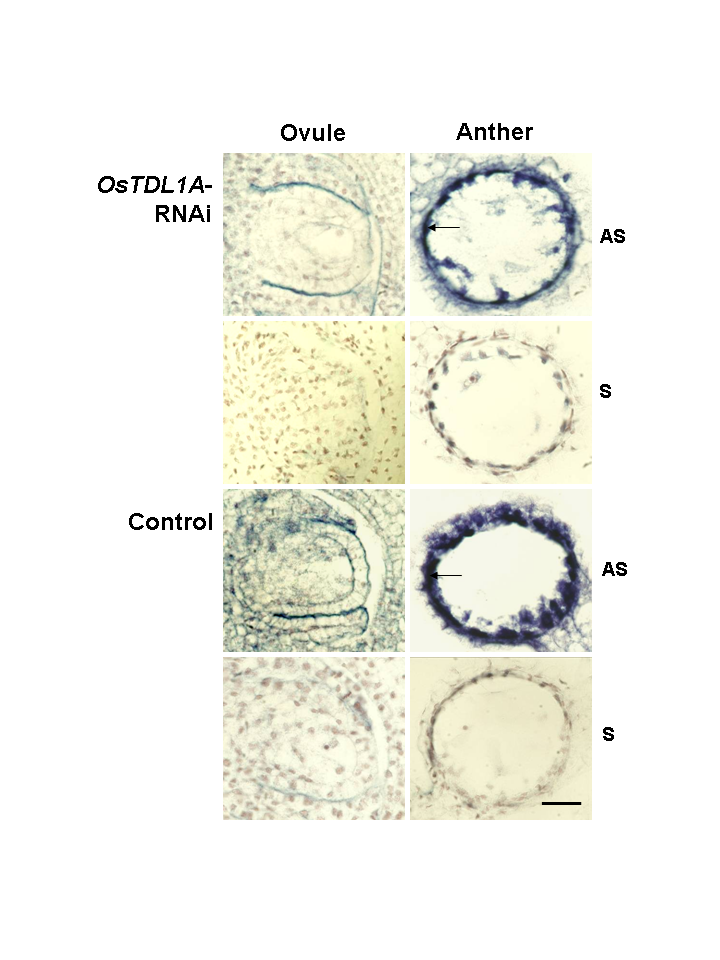

Supplement: Figure S2 — Use of RNA in situ hybridization to detect OsTDL1A transcripts in the ovule and anther of 3 mm spikelets (stage of maximum meiosis) of non-transgenic plants (control) and T2 plants of OsTDL1A-RNAi line #4363. [file tpj0054-0375-SD3.tif]

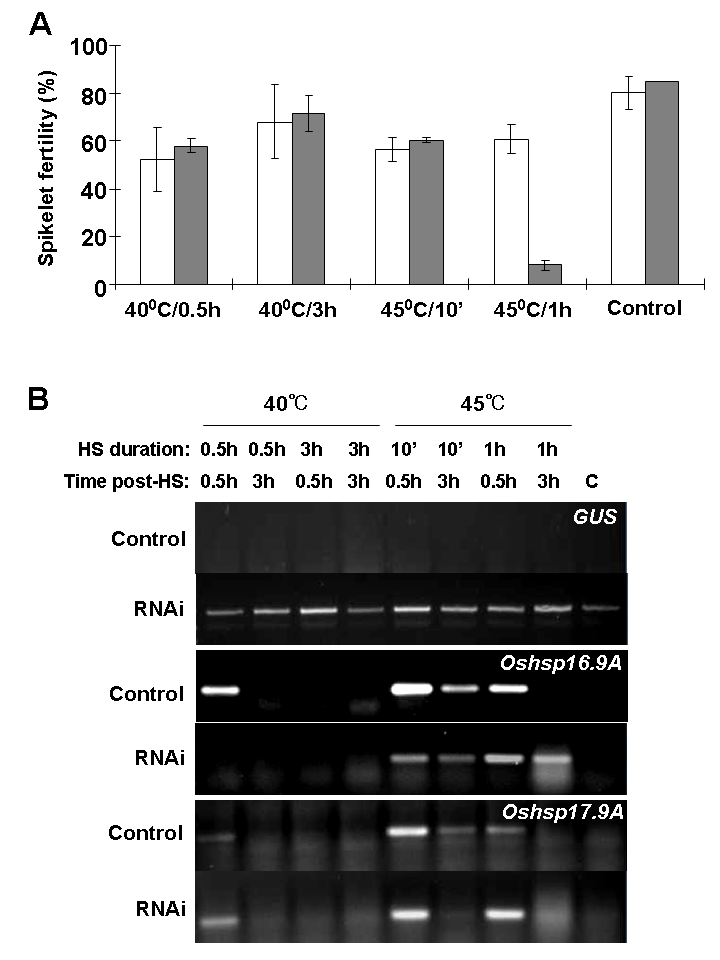

Supplement: Figure S3 — Effect of heat shock (HS) at the booting stage on spikelet fertility and gene expression in cv Nipponbare (control) and T2 plants of OsTDL1A-RNAi line #4363. [file tpj0054-0375-SD4.tif]
